# Supplementary material for: Bayesian refinement of protein structures and ensembles against SAXS data using molecular dynamics
Source: PLoS Comput Biol. 2017 Oct 18;13(10):e1005800. doi: 10.1371/journal.pcbi.1005800 (PMC5662244; doi:10.1371/journal.pcbi.1005800)
Supplement: S1 Archive — (BZ2) [file pcbi.1005800.s011.tar.bz2 › gromacs_4.62_bayesian_saxs_refi/share/html/online/g_dyndom.html]

g\_dyndom


| g\_dyndom Main Table of Contents |

**VERSION 4.6  
Sat 19 Jan 2013**

---

### Description

g\_dyndom reads a .pdb file output from DynDom
(http://www.cmp.uea.ac.uk/dyndom/).
It reads the coordinates, the coordinates of the rotation axis,
and an index file containing the domains.
Furthermore, it takes the first and last atom of the arrow file
as command line arguments (head and tail) and
finally it takes the translation vector (given in DynDom info file)
and the angle of rotation (also as command line arguments). If the angle
determined by DynDom is given, one should be able to recover the
second structure used for generating the DynDom output.
Because of limited numerical accuracy this should be verified by
computing an all-atom RMSD (using g\_confrms) rather than by file
comparison (using diff).

The purpose of this program is to interpolate and extrapolate the
rotation as found by DynDom. As a result unphysical structures with
long or short bonds, or overlapping atoms may be produced. Visual
inspection, and energy minimization may be necessary to
validate the structure.

### Files

| option | filename | type | description |
| --- | --- | --- | --- |
| **-f** | dyndom.pdb | Input | Protein data bank file |
| **-o** | rotated.xtc | Output | Trajectory: xtc trr trj gro g96 pdb |
| **-n** | domains.ndx | Input | Index file |

### Other options

| option | type | default | description |
| --- | --- | --- | --- |
| **-[no]h** | bool | no | Print help info and quit || **-[no]version** | bool | no | Print version info and quit || **-nice** | int | 0 | Set the nicelevel || **-firstangle** | real | 0 | Angle of rotation about rotation vector || **-lastangle** | real | 0 | Angle of rotation about rotation vector || **-nframe** | int | 11 | Number of steps on the pathway || **-maxangle** | real | 0 | DymDom dtermined angle of rotation about rotation vector || **-trans** | real | 0 | Translation (Angstrom) along rotation vector (see DynDom info file) || **-head** | vector | 0 0 0 | First atom of the arrow vector || **-tail** | vector | 0 0 0 | Last atom of the arrow vector |

---

http://www.gromacs.org  
gromacs@gromacs.org
